# Loss of power by degree of heaping in covariables

**DOI:** 10.1186/s12874-026-03043-4

**Published:** 2026-10-17

**Authors:** Matthias Becher, Willi Sauerbrei, Heiko Becher

**Affiliations:** 1 Institute of Biometry and Clinical Epidemiology, Charité – University Medical Center Berlin, corporate member of Freie Universität Berlin and Humboldt-Universität zu Berlin, Charitéplatz 1, 10117 Berlin, Germany; 2 Institute of Medical Biometry and Statistics, Faculty of Medicine and Medical Center, University of Freiburg, Stefan-Meier-Straße 26, 79104 Freiburg, Germany; 3 Heidelberg Institute of Global Health, University Hospital Heidelberg, Im Neuenheimer Feld 130.3, 69120 Heidelberg, Germany

**Keywords:** Heaping, Data blurring, Power, Bias, Epidemiology

## Abstract

Heaping or digit preference is frequently occurring in health research data as well as in deliberate data masking. We investigate the associated loss of power associated by degree of heaping by simulation and illustrate the effect with a real dataset. Moderate heaping leads to little power loss and may be tolerable. However, heaping in broad categories induces major power loss and should be avoided.

## Background

In health research, a common source of information on exposures is the interview of study participants. There are various strategies to obtain the information as precisely as possible. Among the option are: (i) to ask directly for the variable of interest, e.g. the exact age when a certain event occurred, (ii) by giving predefined categories, e.g. various age ranges, or (iii) simply asking a binary question, e.g. occurrence of an event before a certain age yes/no. If asked directly, the response is often heaped, meaning that there is an answering preference of round numbers. In (ii), the heaping is inherent in the questioning, and (iii) represents the extreme form of heaping with two categories only. Another term often used in literature for “heaping” is digit preference (e.g.Pickering, [1]; [2]). 

In a previous analysis, we investigated the bias of the regression coefficient when a covariable is heaped [3]. In that example we used data from the NAKO, a large cohort study performed in Germany [4]. The variable of interest was age when hypertension was diagnosed, and we observed heaping for this variable. The heaping in the age variable appeared visually high, however the percentage of heaped observations was 12.6% only. We showed with a simulation study that the bias is small unless the proportion of study participants which give heaped information is very large and the heaping is in broad categories. We concluded that in this cohort study the bias was small compared to other possible sources of bias and therefore can be neglected [3]. We did not, however, investigate the loss of power, which is associated with this kind of information reduction.

Discussions on data protection and related legislation have led to restrictions being placed on the sharing of data for research purposes. In particular, the aim is to ensure that it is not possible to re-identify individuals. One consequence of this, for example in the German National Cohort, is that a person’s exact age is only disclosed in justified exceptional cases; as a rule, only their placement within 10-year age groups is provided – in other words, a post-hoc heaping is introduced. (https://nako.de/en/participating/mynako/)

Some research has been done regarding heaping. However, most research focused on either quantifying the degree of heaping [5, 6] or investigating methods to deal with heaped data [7, 8]. Denic et al., [9] investigate the quality of age data in developing countries. They find many age endings with 0 and 5 and state that the heaped data may bias results and weaken the power of a study, but they do not investigate it. Nietert et al., [10] investigated electronic medical records from 85 practices around the United States. A number ending with zero was found in 44.6% and 47.5% for systolic and diastolic blood pressure. In a secondary data analysis, [11] included 852 nutrition surveys and assessed the normality of the Mid-Upper Arm Circumference (MUAC) of children from 6 to 59 months. There were 533 surveys which showed departure from normal distributions, 294 (55.2%) of them showed high digit preference.

Despite its relevance in practice, little research has been done quantifying the effect of heaping. Augustin and Wolff, [12] and [7] looked at the resulting bias from heaping. However, [12] focused specifically on the Weibull model and [7] used inferred data to quantify the bias. Heaping occurs also in many other areas, e.g. in census and other surveys, and in blurring data to avoid identifiability of individuals with a so-called bucketing function [13].

Several approaches have been proposed to estimate this bias, although their applicability depends strongly on the type of data and the underlying model. For example, Jung et al., [8] propose a fuzzy heaping model for heaped count data, while Bar and Lillard (2011) develop a model-based approach to estimate the extent of heaping in retrospectively reported event data.

Although heaping differs from classical measurement error, some methods developed to address measurement error are probably transferable to mitigate biases introduced by heaping. A comprehensive discussion of measurement error and correction methods is provided in a two-part paper by the STRATOS initiative’s topic group “Measurement Error and Misclassification” [14, 15].

Regarding the application of these methods, the authors conclude in Part I: “However, our impression is that even when such quantitative information is available, the adjusted analyses are not being performed.” This observation likely also applies to biases arising from heaping.

In this paper, we investigate the loss of power for increasing degree of heaping which to the best of our knowledge has not yet been considered. Dichotomizing a continuous covariable can be considered as the extreme form of heaping which is still often done in the analysis of epidemiological and clinical data although it is known for decades that dichotomizing a continuous variable introduces several severe issues and should be avoided [16–19].

In this paper we demonstrate the loss of power induced by heaping by means of a simulation study in the context of the logistic regression model. For practical illustration we use data from a case-control study on stroke [20].

## Methods

We consider a logistic model as $\:logit\:P(Y=1|X,Z)=a+X\beta\:+Z\gamma\:$. Here Y is the binary outcome variable, X is a covariate matrix with continuous variables subject to heaping, Z is a matrix with other covariates, as is the case in the data example in Sect.  4, and β and γ are the corresponding vectors of coefficients. The covariates in X may be heaped to a degree which is denoted by $\:\delta\:\in\:N$, meaning that any number in X is rounded to the nearest multiple of the rounding value δ.

To estimate the effect of heaping on the power we conducted a simulation study. We consider a simple logistic regression model with outcome $\:Y\in\:\left\{\mathrm{0,1}\right\}$, no covariates Z, and one normal distributed covariate $\:X\sim\:N\left(-\mu\:,\:{\sigma\:}^{2}\right)\:\mathrm{for}\:Y=0\:$and $\:N\left(\mu\:,\:{\sigma\:}^{2}\right)\:\mathrm{for}\:Y=1$ representing a continuous risk factor for the outcome. Table 1 gives an overview of the parameter values. The distribution of the covariable X was chosen such that including X untransformed (effect is linear) into the logistic regression model is correct. The theoretical regression coefficient is then obtained from the equation $\:\beta\:=\frac{2\mu\:}{{\sigma\:}^{2}}$ (see [21].

**Table 1:** Parameters for the simulation study

Parameter	Values	Β	E(X|X < 0)
$\:\left(\mu\:,{\sigma\:}^{2}\right)$	(0.5, 100)	0.01	-7.99
	(0.5, 200)	0.005	-11.29
	(1.0, 100)	0.02	-8.02
	(1.0, 200)	0.01	-11.31
	(1.5, 100)	0.03	-8.07
	(1.5, 200)	0.015	-11.35
n	250, 500, 750		
$\:\delta\:$	0, 1, 5, 10, 20, D (dichotomized)		

For each combination of the parameters and for each chosen sample sizes n for Y = 0 and Y = 1 (i.e. total sample size 2n), we simulated data sets. Then all values for X were rounded according to δ (complete heaping). A logistic regression was performed and the p-value of the coefficient for X was stored. For the dichotomization we assigned to each observation the expected value of the Variable X given X < 0 or X > 0. This was done to achieve an identical expected regression coefficient as for the original data. It was calculated according to the formula

$$\begin{array}{lll} E\left(X|X < 0\right) & = & E\left(X|X < 0,Y=0\right)P\left(Y=0|X < 0\right)\\&&+\,E\left(X|X < 0,Y=1\right)\,P\left(Y=1|X < 0\right)\\ & = & \left[\mu_0-\sigma\,\left(\frac{\varphi\left(-\frac{\mu_0}{\sigma}\right)}{\phi\left(-\frac{\mu_0}{\sigma}\right)}\right)\right]\,P\left(Y=0|X < 0\right)\\&&+\left[\mu_1-\sigma\left(\frac{\varphi\left(-\frac{\mu_1}{\sigma}\right)}{\phi\,\left(-\frac{\mu_1}{\sigma}\right)}\right)\right]\,P\left(Y=1|X<0\right)\end{array},$$

where φ and ϕ denote the density resp. distribution function of the standard normal distribution. The resulting values for the different parameter combinations are given in the last column of Table 1.

This process was repeated M = 100,000 times. The choice of the number of replications in the simulation was chosen such that the Monte Carlo standard error of the estimate is smaller than 1% of the mean based on the recommendations of [22]. The Monte Carlo standard error of the estimate is $\:\sqrt{\frac{1}{M(M-1)}{\sum\:}_{m=1}^{M}{\left({\beta\:}_{\mathrm{m}}-\stackrel{-}{\beta\:}\right)}^{2}}$, where $\:{\beta\:}_{m}$ is the simulated parameter in the m-th simulation run. The mean of the estimates $\:{\beta\:}_{m},\:\stackrel{-}{\beta\:}$, and the p-values $\:{p}_{m},\:\stackrel{-}{p}$, were returned as the result.

We consider the change of $\:\stackrel{-}{p}$ with increasing degree of heaping as the suitable measure for power loss. See A.2 for a brief overview of the study following the ADEMP structure [22].

We further calculated the effective sample size after heaping as a percentage of the original sample size. To do that we estimated the required sample size to achieve the observed power after heaping, following the approach by [23].

## Results

In Table 2 we present the results of the simulation. For each parameter combination we give the mean of the regression estimates $\:\stackrel{-}{\beta\:}$, the mean p-values. The corresponding simulation standard errors of the p-values were smaller than 10^− 4^ in all cases and are omitted from this table for better readability.

**Table 2:** Loss of power by degree of heaping δ - Results of the simulation study

	parameter combination ($\:n,\boldsymbol{\mu\:},\:{\boldsymbol{\sigma\:}}^{2})$											
	(250, 0.5, 100)		(250, 0.5, 200)		(250, 1, 100)		(250, 1, 200)		(250, 1.5, 100)		(250, 1.5, 200)	
$\:\boldsymbol{\delta\:}$	$\:\stackrel{-}{\beta\:}$	$\:\stackrel{-}{p}$	$\:\stackrel{-}{\beta\:}$	$\:\stackrel{-}{p}$	$\:\stackrel{-}{\beta\:}$	$\:\stackrel{-}{p}$	$\:\stackrel{-}{\beta\:}$	$\:\stackrel{-}{p}$	$\:\stackrel{-}{\beta\:}$	$\:\stackrel{-}{p}$	$\:\stackrel{-}{\beta\:}$	$\:\stackrel{-}{p}$
0	0.010	0.337	0.005	0.410	0.020	0.108	0.010	0.229	0.030	0.018	0.015	0.090
1	0.010	0.337	0.005	0.410	0.020	0.108	0.010	0.229	0.030	0.018	0.015	0.090
5	0.010	0.340	0.005	0.411	0.020	0.111	0.010	0.231	0.030	0.019	0.015	0.091
10	0.009	0.348	0.005	0.413	0.019	0.121	0.010	0.236	0.028	0.023	0.015	0.096
20	0.008	0.373	0.004	0.422	0.015	0.158	0.009	0.256	0.023	0.040	0.013	0.114
D^*^	0.010	0.388	0.005	0.440	0.020	0.186	0.010	0.302	0.030	0.057	0.015	0.165
	parameter combination ($\:n,\boldsymbol{\mu\:},\:{\boldsymbol{\sigma\:}}^{2})$											
	(500, 0.5, 100)		(500, 0.5, 200)		(500, 1, 100)		(500, 1, 200)		(500, 1.5, 100)		(500, 1.5, 200)	
0	0.010	0.227	0.005	0.336	0.020	0.025	0.010	0.106	0.030	0.001	0.015	0.017
1	0.010	0.228	0.005	0.336	0.020	0.025	0.010	0.106	0.030	0.001	0.015	0.017
5	0.010	0.231	0.005	0.337	0.020	0.026	0.010	0.108	0.030	0.001	0.015	0.018
10	0.009	0.242	0.005	0.341	0.019	0.031	0.010	0.113	0.028	0.001	0.015	0.020
20	0.008	0.277	0.004	0.356	0.015	0.051	0.009	0.131	0.023	0.004	0.013	0.027
D^*^	0.010	0.301	0.005	0.388	0.020	0.071	0.010	0.183	0.030	0.007	0.015	0.056
	parameter combination ($\:n,\boldsymbol{\mu\:},\:{\boldsymbol{\sigma\:}}^{2})$											
	(750, 0.5, 100)		(750, 0.5, 200)		(750, 1, 100)		(750, 1, 200)		(750, 1.5, 100)		(750, 1.5, 200)	
0	0.010	0.157	0.005	0.278	0.020	0.006	0.010	0.052	0.030	0.000	0.015	0.004
1	0.010	0.157	0.005	0.278	0.020	0.006	0.010	0.052	0.030	0.000	0.015	0.004
5	0.010	0.160	0.005	0.280	0.020	0.007	0.010	0.053	0.029	0.000	0.015	0.004
10	0.009	0.171	0.005	0.285	0.019	0.009	0.010	0.056	0.028	0.000	0.014	0.005
20	0.008	0.209	0.004	0.302	0.015	0.018	0.009	0.071	0.023	0.000	0.013	0.007
D^*^	0.010	0.237	0.005	0.344	0.020	0.029	0.010	0.115	0.030	0.001	0.015	0.021

^*^D – Dichotomization at the median

We observe that with increasingly coarse heaping the p-value gets more and more distorted. For a moderate heaping (up to rounding to the next multiple of 5) the loss of power is mild. As expected, the largest power reduction is for the extreme case when the variable is dichotomized. Note that the change in $\:\stackrel{-}{\beta\:}$ with increasing degree of heaping describes the bias through heaping. The simulation shows that a bias is apparent only if the heaping is rather coarse ($\:\boldsymbol{\delta\:}=10$ or $\:\boldsymbol{\delta\:}=20$ ). There is no bias in the case of dichotomization if the expected value of X given < 0 resp. >0 is assigned to X. Note also that the p-value in case of dichotomization is independent of the assigned value. Thus, if the assigned values are 0 and 1, as commonly done for a dichotomized variable, the p-value remains unchanged.

**Fig. 1:**
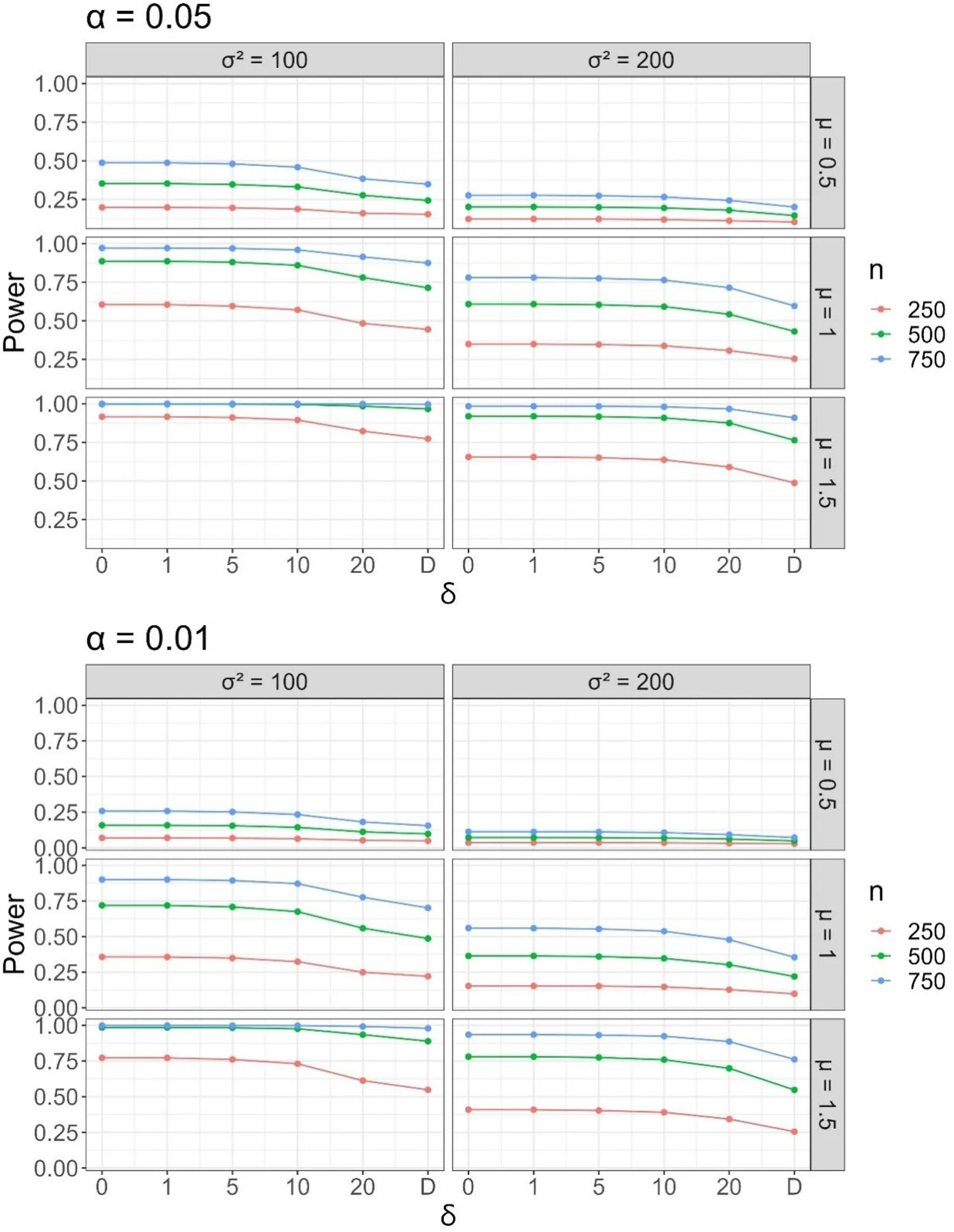
The simulated power $\:P(p<\alpha\:)$ for the different simulation scenarios and α-levels. The power is shown for different levels of heaping, ranging from 0 (no heaping) to D (dichotomization). The colors correspond to the different sample sizes per group

Figure 1 shows the simulated power for our scenarios across the different rounding values as the proportion of observations with p-values smaller than 0.05 or 0.01 by degree of heaping. It further supports the observations made in Table 2. Loss of power is almost negligible up to heaping to the nearest multiple of 10. As expected, dichotomization is generally associated with a relevant drop in power in all scenarios.

**Fig. A.1:**
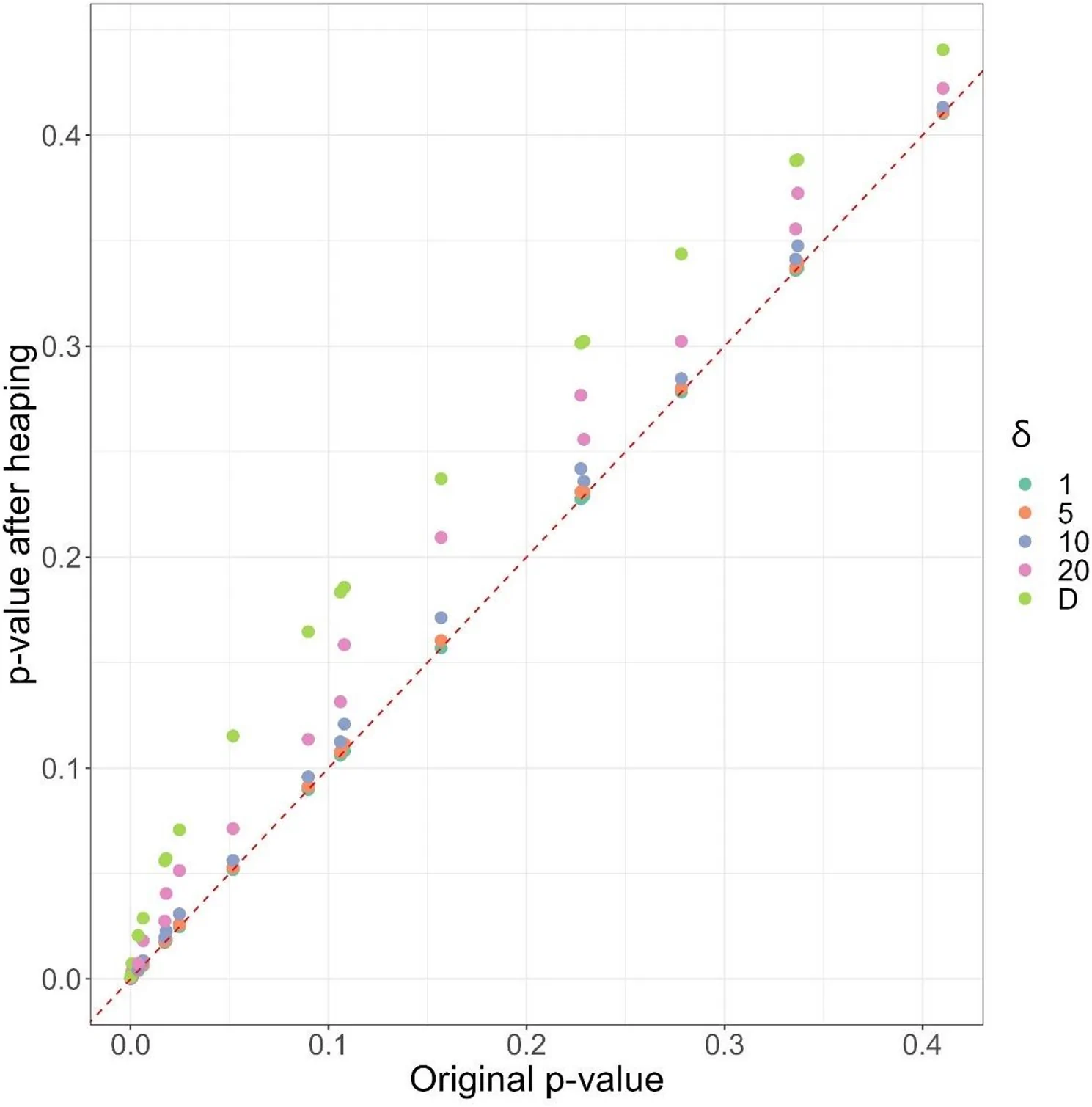
Simulation result: Original mean p-value vs. mean p-value after heaping. δ – degree of heaping (D – Dichotomization).

Figure A.1 in the appendix shows the plot of the original p-values vs. the p-values as obtained from various degrees of heaping.

**Fig. 2:**
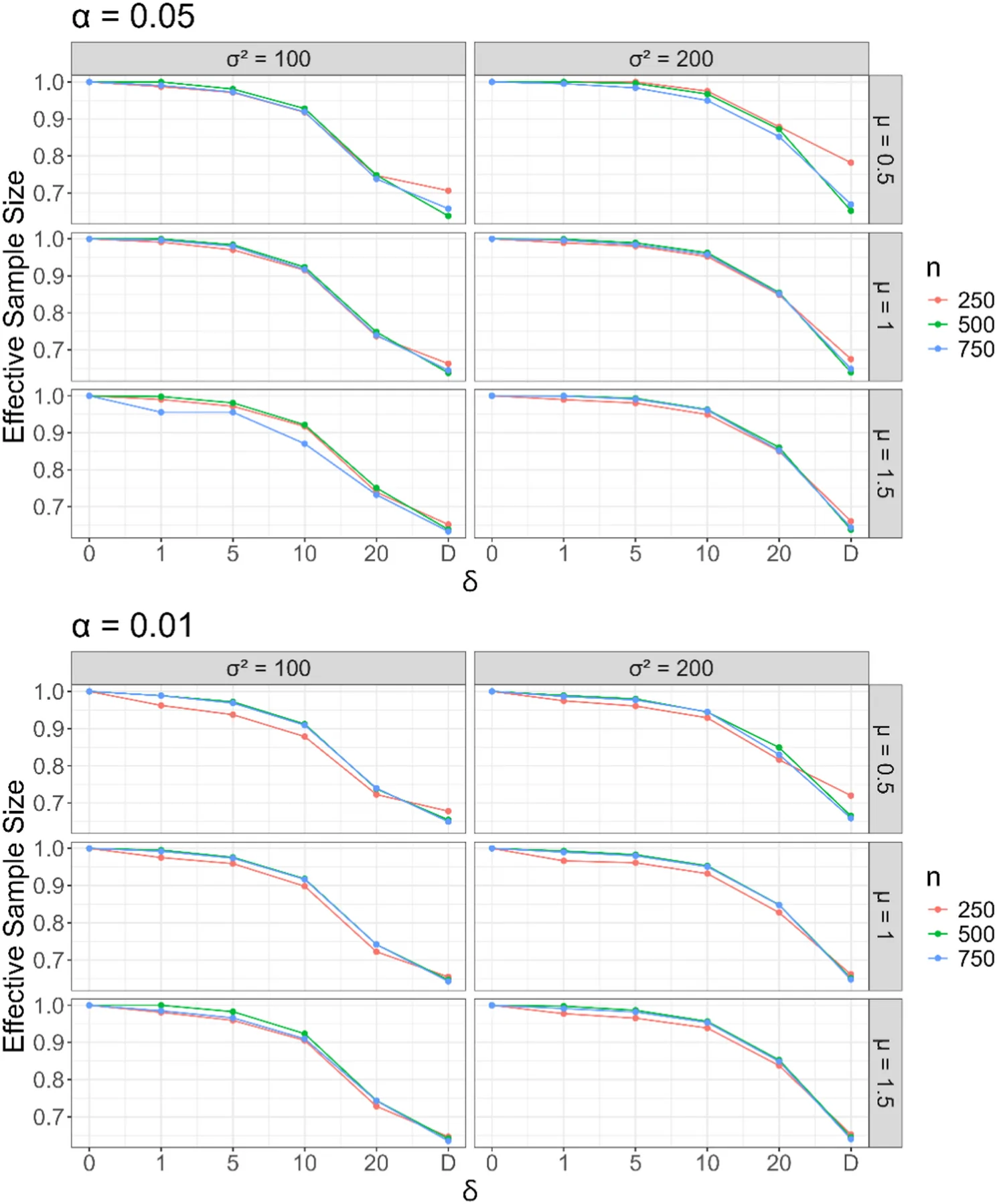
Effective Sample Size as a proportion of the original sample size for different levels of heaping, ranging from 0 (no heaping) to D (dichotomization). The colors correspond to the different sample sizes per group

Figure 2 shows the effective sample size after heaping as a proportion of the original sample size for α = 0.05 and α = 0.01. For moderated levels of heaping the effective sample size remains close to the actual sample size, mirroring the results for the power. However, for coarser heaping the effective sample size drops of noticeably, reaching around 70% of the original sample size for dichotomization.

## Data example

We use data from a case-control study on stroke with the primary aim to investigate the effect of dental infections on stroke risk [20]. The study was performed in the years 1998–2000 in Heidelberg, Germany, and included 303 incident cases of ischemic stroke (208 males, 95 females) and 468 controls (302 males, 166 females). As main risk factors smoking and alcohol consumption were considered. In this analysis we excluded tea-totallers and never smokers. The analysis is then based on 181 cases and 221 controls. We use years of smoking and total amount of alcohol as quantitative variables. Assuming linear effects for both variables, the logistic regression analysis allows estimating the joint odds ratio function for the two main risk factors of stroke among those who were smokers and who consumed alcohol. Age (in years, linear effect) and sex are included as confounders. For both continuous risk variables the effect of heaping on the estimates and the p-values was investigated. As there was little heaping in the original data, we induced heaping for this analysis. Smoking was heaped by rounding to the next value of multiples of 2, 5, 10, and dichotomising at the median. The cutpoint here was 29 years. For alcohol we used 50, 100, 200, 500 and dichotomising at the median. The cutpoint here was 115.012 L. When dichotomizing, we assigned the observed mean given above resp. below the cutpoint to each individual regardless of case-control status. The means are 17.00 years and 39.68 years for smoking, and 37.75 L and 557.21 L for alcohol.

**Fig. 3:**
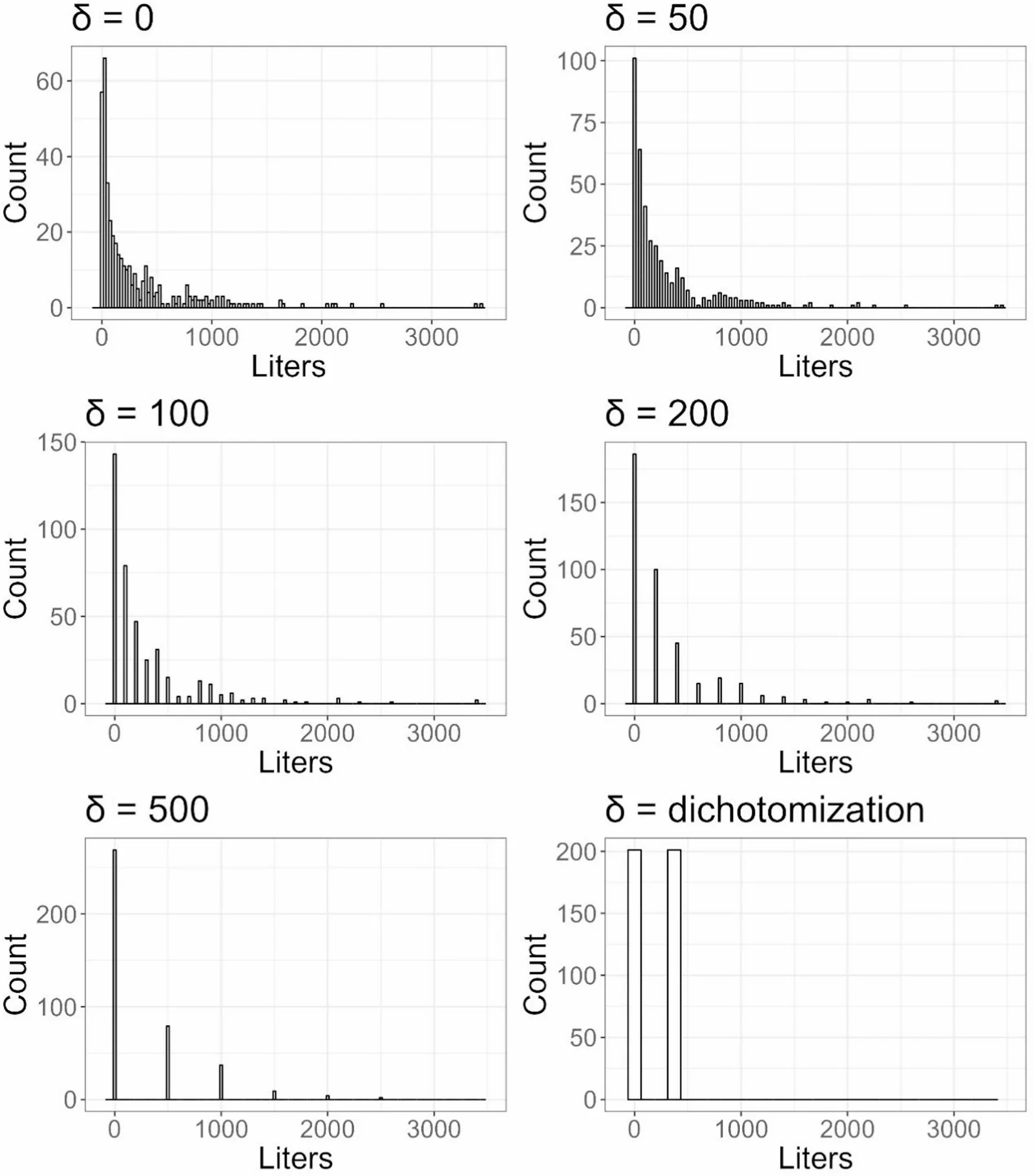
Induced heaping for alcohol

**Fig. 4:**
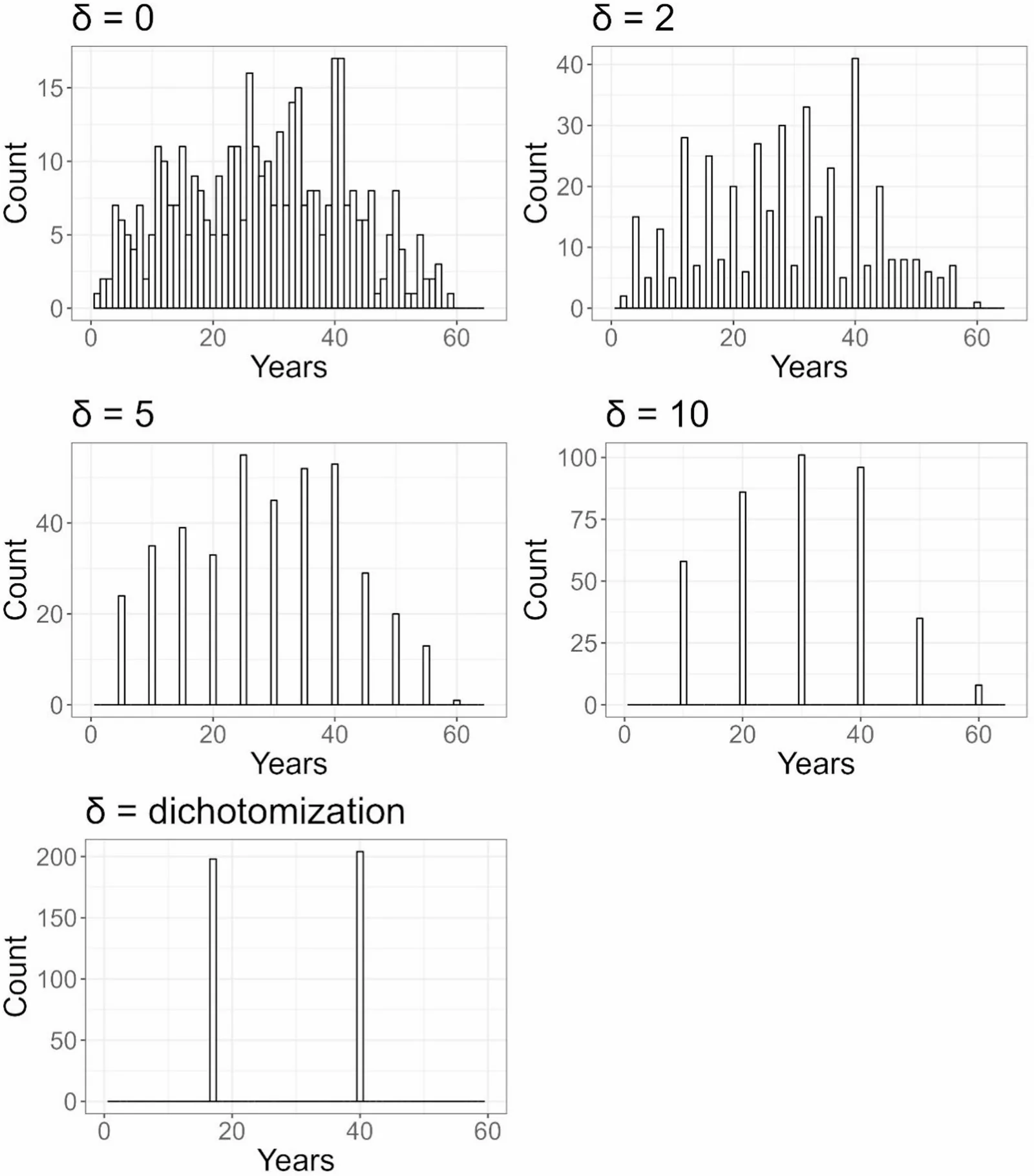
Induced heaping for smoking

Figures 3 and 4 show the distribution of after heaping to the different rounding values for alcohol and smoking respectively. For dichotomization each value smaller than or equal to the median was set to the 25%-quantile and each value larger to the 75%-quantile.

The distribution of the alcohol variable is heavily right skewed. In contrast, the distribution of smoking is fairly symmetric. The correlation between the two variables is almost zero $\:\left({\rho\:}_{spearman}=0.0017\right).$

In Table 3 we present the p-values from the logistic regression model for all heaping combinations. The original data give p-values of 0.049 for alcohol and 0.041 for smoking. The p-values gradually increase to the maximum of 0.88 and 0.12 for alcohol and smoking, respectively, when both variables are dichotomized. These results are largely in line with the results from the simulation.

**Table 3:** Regression coefficient estimates and p-Values for alcohol and smoking for different degrees of heaping

		Degree of heaping for alcohol ($\:{\delta\:}_{alc}$)					
	$\:{\beta\:}_{alc}\mathrm{*}{10}^{3}\:\left(p\right)$ $\:{\beta\:}_{smo}\mathrm{*}10\:\left(p\right)$	Original	50	100	200	500	D^*^
Degree of heaping for smoking ($\:{\delta\:}_{smo}$)	Original	0.47 (0.049) 0.16 (0.041)	0.48 (0.043) 0.16 (0.041)	0.44 (0.060) 0.16 (0.042)	0.45 (0.051) 0.16 (0.041)	0.26 (0.211) 0.16 (0.046)	0.12 (0.841) 0.15 (0.052)
	2	0.47 (0.049) 0.16 (0.043)	0.48 (0.043) 0.16 (0.041)	0.44 (0.060) 0.16 (0.043)	0.45 (0.051) 0.16 (0.041)	0.26 (0.211) 0.16 (0.046)	0.12 (0.843) 0.15 (0.054)
	5	0.46 (0.050) 0.15 (0.056)	0.48 (0.045) 0.15 (0.056)	0.44 (0.062) 0.15 (0.057)	0.45 (0.053) 0.15 (0.056)	0.26 (0.216) 0.15 (0.061)	0.12 (0.847) 0.14 (0.069)
	10	0.46 (0.050) 0.14 (0.061)	0.48 (0.045) 0.14 (0.061)	0.44 (0.062) 0.14 (0.063)	0.45 (0.053) 0.14 (0.061)	0.26 (0.219) 0.14 (0.067)	0.11 (0.856) 0.14 (0.075)
	D^*^	0.47 (0.047) 0.16 (0.092)	0.48 (0.042) 0.16 (0.091)	0.45 (0.057) 0.16 (0.094)	0.46 (0.050) 0.16 (0.093)	0.27 (0.204) 0.15 (0.103)	0.09 (0.884) 0.15(0.124)

^*^D – Dichotomization at the median

Furthermore, we can see that while the p-value generally increases with stronger heaping there are exceptions.

For the alcohol variable, the change in the regression estimate with a high degree of heaping is remarkable. When dichotomizing the variable, the effect almost disappears.

## Discussion

Heaping or digit preference is common in observational data and can be considered as a special kind of measurement error. While the effect of heaping in terms of both precision and bias may often be small, it can lead to relevant problems such as distorted effect estimates, false decisions concerning inclusion of variables in a multivariable model and therefore seriously wrong conclusions, in particular when heaping is close to dichotomization as the extreme case of heaping. In this study we investigated the effect of different levels of heaping, and the associated information loss, on power. To that end we have conducted a simulation study. The results clearly show that an increasingly coarse heaping leads to higher p-values and thus to a loss in power. As expected, dichotomization leads to maximal power loss.

Although several critical issues are known for decades [16, 18], dichotomization of continuous variables is still very popular in many areas of research. For example, continuous biomarkers are often dichotomized to assess whether they have a prognostic effect [24] and in randomized trials interactions with continuous variables (e.g. age) are usually investigated by selecting a small number of values to create two, three or four subgroups [25]. In the latter context, increased type I error and loss of power was shown in simulation studies [26, 27].

It is important to note, that the simulation results represent a scenario in which all data is heaped. In contrast, in epidemiologic studies as in our data example, researchers are likely to encounter partial heaping which would reduce the associated power loss. In case of intended data blurring, heaping applies to all observations, and then the simulation results are directly applicable.

Our results may also be relevant for statistical disclosure control (SDC) and therefore for making data publicly available such that they are sufficiently anonymous and still allow an analysis with almost full power. SDC, discussed for decades, seeks to modify statistical data so that they can be published without giving away confidential information that can be linked to specific respondents. The challenge for SDC is to achieve this modification with minimum loss of the detail and accuracy sought by database users. The aforementioned application of a bucketing function which is not too broad could be suitable [13]. Further discussions on de-identification of individuals in other research areas in the context of data sharing is given e.g. in [28]. Here they use the term „microaggegation“ for heaping. They conclude that “This technique is very useful for de-identifying data where publicly available data that can be linked exist. “. No statement on the effect on the results are provided. We also conclude that the data protection policy of the NAKO cohort study is tolerable since the power loss induced by providing age in 10-year age groups is small. On the other hand, a re-identification is made considerably more difficult.

Our approach – heaping - considers a specific deviation from the true values. The results are, as expected, in line with established knowledge regarding information loss arising from measurement error, discretization, grouping, and dichotomization of continuous variables.

In our paper, we considered a binary outcome and a logistic regression model. We assume that our results generally hold for regression models [14, 29]. In order to further support this assumption, in appendix A.3 we report the results from the simulation based on a linear model. Here we use an equation which may resemble the relation between age and systolic blood pressure [30]. The results are totally in line with the results from the logistic regression.

We also illustrated the effect of heaping with real data from a case-control study. Overall, the results from the data example match the ones from the simulation. However, they also showed that the relationship between heaping and p-value is not strictly monotonous and depend on the distributions of the variables and their correlation. While the power generally decreases with increasing heaping, there were single instances in which the p-value increased. This is likely due to the data structure of this particular data set and means that in real data the effect can be unpredictable.

In summary, our results show that mild levels of heaping, no matter whether they result from direct responses from a fraction of study participants, or from deliberate data blurring, are acceptable from a statistical point of view, since it causes only negligible and tolerable power loss. According to our experience, this is the common case in practice. Extreme cases of heaping, however, such as dichotomization or rounding such that very few distinct levels remain should be avoided.

## Data Availability

The datasets used and/or analysed during the current study are available from the corresponding author on reasonable request.

## Appendix

**Table A.2:** Brief overview of the study following the ADEMP structure

Aims	To investigate the power loss associated with heaping of a variable when assessing its effect on an outcome via logistic regression To estimate the change of the mean *p*-value caused by heaping. For two continuous variables, investigate whether the relationship between heaping and *p*-values is strictly monotonous.
Data	We simulated data for 6 different combinations of effects and variances. We used three different sample sizes. See Table 1 for details. To investigate the effect of heaping for two continuous variables, we used data from a case-control study about the effect of dental infections on stroke risk (181 cases, 221 controls)
Estimand/target of analysis	In the context of logistic regression, investigate the effect of heaping on the change of p-values and on power.
Methods	Simulation study to estimate the effect of heaping for 5 pre-specified heaping categories from very weak to dichotomization. Simulate data and perform a logistic regression to obtain an estimate for beta and an associated p-value In the case-control study, use logistic regression to derive estimates for two continuous variables and investigate the effect of heaping.
Performance measures	Compare the mean estimated effects and mean p-value of the original data with the results of the different levels of heaping. For a = 0.01 and a = 0.05 estimate the effect of heaping on the loss of power.

**Table A.3:** Small simulation of a linear regression model and heaping of a continuous covariable

Degree of heaping	Mean *p*-value	Standard deviation *p*-value	Relative sample size
0	0.095	0.160	1
1	0.095	0.161	0.999
5	0.097	0.164	0.975
10	0.100	0.166	0.950
20	0.111	0.182	0.857
D	0.159	0.218	0.599

Model: $$y=\alpha+\beta \,x+\epsilon\,with\,\alpha =100;\beta\:=0.5;\epsilon\sim N\left(0,25\right);x\sim {Unif}\left(20,80\right);n=50;400\,simulation\,runs$$

The mean p-values increase with increasing level of rounding from 0.095 to 0.159 which corresponds to a decrease of the effective sample size from 100% to 59.7%. The results are in line with the results from the primary simulation study in Fig. 2.

## References

[1] Pickering R. Digit preference in estimated gestational age. Stat Med. 1992;11(9):1225–38. 10.1002/sim.4780110908 1509222

[2] de Lusignan S, Belsey J, Hague N, Dzregah B. End-digit preference in blood pressure recordings of patients with ischaemic heart disease in primary care. J Hum Hypertens. 2004;18(4):261–5. 10.1038/sj.jhh.1001663 15037875

[3] Jaeschke L, Becher M, Velásquez IM, Ahrens W, Bächle C, Baurecht H, Fricke J, Greiser KH, Günther K, Heier M, Karch A, Kluttig A, Krist L, Leitzmann M, Michels K, Mikolajczyk R, Peters A, Schipf S, Völzke H, Becher H. The bias from heaping on risk estimation: effect of age at diagnosis of hypertension on risk of subsequent cardiovascular comorbidities. Ann Epidemiol. 2022;74:84–96. 10.1016/j.annepidem.2022.07.012 35940393

[4] Peters A, de GNCChndgn, Peters A, Greiser KH, Göttlicher S, Ahrens W, Albrecht M, Bamberg F, Bärnighausen T, Becher H. Framework and baseline examination of the German National Cohort (NAKO) Peters et al. Eur J Epidemiol. 2022;37(10):1107–24. 10.1007/s10654-022-00890-5 PMC9581448 36260190

[5] Lyons-Amos M, Stones T. Trends in Demographic and Health Survey data quality: an analysis of age heaping over time in 34 countries in Sub Saharan Africa between 1987 and 2015. BMC Res Notes. 2017;10:1–7. 10.1186/s13104-017-3091-x PMC5738749 29262857

[6] Roberts JM, Brewer DD. Measures and tests of heaping in discrete quantitative distributions. J Applied Statistics. 2001;28(7):887–96.

[7] Bar HY, Lillard DR. Accounting for heaping in retrospectively reported event data–a mixture-model approach. Stat Med. 2012;31(27):3347–65. 10.1002/sim.5419 PMC3518030 22733577

[8] Jung H-Y, Choi H, Park T. Fuzzy heaping mechanism for heaped count data with imprecision. Soft Comput. 2018;22:4585–94.

[9] Denic S, Saadi H, Khatib F. Quality of age data in patients from developing countries. J Public Health. 2004;26(2):168–71. 10.1093/pubmed/fdh131 15284321

[10] Nietert PJ, Wessell AM, Feifer C, Ornstein SM. Effect of terminal digit preference on blood pressure measurement and treatment in primary care. Am J Hypertens. 2006;19(2):147–52. 10.1016/j.amjhyper.2005.08.016 16448884

[11] Frison S, Checchi F, Kerac M, Nicholas J. Is middle-upper arm circumference normally distributed? Secondary data analysis of 852 nutrition surveys. Emerg themes Epidemiol. 2016;13:1–8. 10.1186/s12982-016-0048-9 PMC4855367 27148390

[12] Augustin T, Wolff J. A bias analysis of Weibull models under heaped data. Stat Pap. 2004;45:211–29.

[13] Jayapradha J, Prakash M, Alotaibi Y, Khalaf OI, Alghamdi SA. Heap bucketization anonymity—An efficient privacy-preserving data publishing model for multiple sensitive attributes. IEEE Access. 2022;10:28773–91.

[14] Keogh RH, Shaw PA, Gustafson P, Carroll RJ, Deffner V, Dodd KW, Küchenhoff H, Tooze JA, Wallace MP, Kipnis V. STRATOS guidance document on measurement error and misclassification of variables in observational epidemiology: part 1—basic theory and simple methods of adjustment. Stat Med. 2020;39(16):2197–231. 10.1002/sim.8532 PMC7450672 32246539

[15] Shaw PA, Gustafson P, Carroll RJ, Deffner V, Dodd KW, Keogh RH, Kipnis V, Tooze JA, Wallace MP, Küchenhoff H. STRATOS guidance document on measurement error and misclassification of variables in observational epidemiology: part 2—more complex methods of adjustment and advanced topics. Stat Med. 2020;39(16):2232–63. 10.1002/sim.8531 PMC7272296 32246531

[16] Cohen J. The cost of dichotomization. Appl Psychol Meas. 1983;7(3):249–53.

[17] Farewell VT, Tom BDM, Royston P. The impact of dichotomization on the efficiency of testing for an interaction effect in exponential family models. J Am Stat Assoc. 2004;99(467):822–31.

[18] MacCallum RC, Zhang S, Preacher KJ, Rucker DD. On the practice of dichotomization of quantitative variables. Psychol Methods. 2002;7(1):19. 10.1037/1082-989x.7.1.19 11928888

[19] Royston P, Altman DG, Sauerbrei W. Dichotomizing continuous predictors in multiple regression: a bad idea. Stat Med. 2006;25(1):127–41. 10.1002/sim.2331 16217841

[20] Grau AJ, Becher H, Ziegler CM, Lichy C, Buggle F, Kaiser C, Lutz R, Bültmann S, Preusch M, Dörfer CE. Periodontal disease as a risk factor for ischemic stroke. Stroke. 2004;35(2):496–501. 10.1161/01.STR.0000110789.20526.9D 14707235

[21] Becher H, Lorenz E, Royston P, Sauerbrei W. Analysing covariates with spike at zero: a modified FP procedure and conceptual issues. Biom J. 2012;54(5):686–700. 10.1002/bimj.201100263 22778015

[22] Morris TP, White IR, Crowther MJ. Using simulation studies to evaluate statistical methods. Stat Med. 2019;38(11):2074–102. 10.1002/sim.8086 PMC6492164 30652356

[23] Hsieh FY, Bloch DA, Larsen MD. A simple method of sample size calculation for linear and logistic regression. Stat Med. 1998;17(14):1623–34. 10.1002/(sici)1097-0258(19980730)17:14<1623::aid-sim871>3.0.co;2-s 9699234

[24] Altman DG, Lausen B, Sauerbrei W, Schumacher M. Dangers of using optimal cutpoints in the evaluation of prognostic factors. J Natl Cancer Inst. 1994;86(11):829–35. 10.1093/jnci/86.11.829. 8182763

[25] Eikelboom JW, Wallentin L, Connolly SJ, Ezekowitz M, Healey JS, Oldgren J, Yang S, Alings M, Kaatz S, Hohnloser SH. Risk of bleeding with 2 doses of dabigatran compared with warfarin in older and younger patients with atrial fibrillation: an analysis of the randomized evaluation of long-term anticoagulant therapy (RE-LY) trial. Circulation. 2011;123(21):2363–72. 10.1161/CIRCULATIONAHA.110.004747 21576658

[26] Haller B, Ulm K, Hapfelmeier A. A simulation study comparing different statistical approaches for the identification of predictive biomarkers. Comput Math Methods Med. 2019;2019(1):7037230. 10.1155/2019/7037230 PMC6595324 31312252

[27] Royston P, Sauerbrei W. Interaction of treatment with a continuous variable: simulation study of power for several methods of analysis. Stat Med. 2014;33(27):4695–708. 10.1002/sim.6308 25244679

[28] Shero JA, Swanz AE, Hanson AL, Hart SA, Logan JA. Data Deidentification for data sharing in education and psychological research: importance, barriers, and techniques. AERA open. 2025;11:23328584251352814. 10.1177/23328584251352814 PMC12700633 41394248

[29] Carroll RJ, Ruppert D, Stefanski LA, Crainiceanu CM. Measurement Error in Nonlinear Models: A Modern Perspective, Second Edition (2nd ed). Chapman and Hall/CRC; 2006. 10.1201/9781420010138.

[30] Lawes CM, Hoorn V, S., Rodgers A. Global burden of blood-pressure-related disease, 2001. Lancet. 2008;371(9623):1513–8. 10.1016/S0140-6736(08)60655-8 18456100

